# Complexities, variations, and errors of numbering within clinical notes: the potential impact on information extraction and cohort-identification

**DOI:** 10.1186/s12911-019-0784-1

**Published:** 2019-04-04

**Authors:** David A. Hanauer, Qiaozhu Mei, V. G. Vinod Vydiswaran, Karandeep Singh, Zach Landis-Lewis, Chunhua Weng

**Affiliations:** 1 Department of Pediatrics, University of Michigan, Ann Arbor, MI 48109 USA; 2 School of Information, University of Michigan, Ann Arbor, MI 48109 USA; 3 Department of Learning Health Sciences, University of Michigan, Ann Arbor, MI 48109 USA; 4 Department of Biomedical Informatics, Columbia University, New York, NY 10032 USA

**Keywords:** Lexical variation, Natural language processing, Information retrieval

## Abstract

**Background:**

Numbers and numerical concepts appear frequently in free text clinical notes from electronic health records. Knowledge of the frequent lexical variations of these numerical concepts, and their accurate identification, is important for many information extraction tasks. This paper describes an analysis of the variation in how numbers and numerical concepts are represented in clinical notes.

**Methods:**

We used an inverted index of approximately 100 million notes to obtain the frequency of various permutations of numbers and numerical concepts, including the use of Roman numerals, numbers spelled as English words, and invalid dates, among others. Overall, twelve types of lexical variants were analyzed.

**Results:**

We found substantial variation in how these concepts were represented in the notes, including multiple data quality issues. We also demonstrate that not considering these variations could have substantial real-world implications for cohort identification tasks, with one case missing > 80% of potential patients.

**Conclusions:**

Numbering within clinical notes can be variable, and not taking these variations into account could result in missing or inaccurate information for natural language processing and information retrieval tasks.

## Background

Much of medicine is quantitative, so it is no surprise that numbers and other numerical concepts are found throughout clinical notes. These numbers can appear in information for ages, dates, laboratory results, temporal constraints of clinical events, severity, risk prediction (e.g., odds ratios), rankings, and other expressions of quantity. As more and more hospitals, health systems, and clinics adopt electronic health records (EHRs) [1] there has been a concurrent interest in finding ways to make better and more meaningful use of the data, [2] including those embedded within the free text clinical notes derived from EHRs. This has led to substantial work in the areas of information extraction, natural language processing, [3] and information retrieval [4–6].

There are many challenges for accurately processing and extracting meaning from clinical notes, details of which have been described elsewhere [7, 8]. These challenges include spelling errors, [9] ambiguous abbreviations and acronyms, [10–12] temporal relationships, [13–15] and the use of hedge phrases [16]. While prior authors have noted that variations exist in how numbers and other numerical concepts are recorded, the literature is lacking in illustrative examples of how these may be represented in clinical notes, which is important for developing targeted solutions when constructing robust information extraction systems. As information extraction tasks become more mainstream, ensuring that all relevant data are accurately identified will become increasingly important. Therefore, it is essential to understand the types of variability and mistakes that can appear in EHR clinical notes.

In this work, we sought to characterize and highlight several unusual characteristics of clinical notes that may be overlooked in typical information extraction tasks. Namely, we sought to quantify the variability in how numbers and numerical concepts are represented in the clinical notes, focusing primarily on deviations from typical Arabic number usage as well as other ways in which numbers were used inappropriately or described invalid scenarios such as biologically implausible ages. Many illustrative examples are provided to highlight the magnitude of the issue. We also quantified the impact of these variations on cohort identification tasks using 10 scenarios in which patient cohorts were identified using Arabic or Roman numerals. The results of this work may be of interest to those who need to extract numeric expressions from clinical notes, and especially to those who work in the area of clinical research informatics for EHR phenotyping and cohort identification [17–21].

## Methods

### Clinical setting

This study took place at Michigan Medicine, an integrated, tertiary care provider comprised of 3 hospitals and 40 outpatient locations in Southeastern Michigan. Michigan Medicine implemented a homegrown EHR in 1998 which was used until its replacement by a vendor system (Epic, Epic Systems, Verona, WI). Epic was implemented in the ambulatory care setting in August 2012, followed by the inpatient setting in June 2014. Approaches to creating clinical notes (i.e., clinical documents) in both systems include typing as well as dictation/transcription. The clinical notes (e.g., progress notes, discharge summaries, pathology reports, radiology reports, etc.) are primarily free text. Notes are created by various clinicians and health professionals including physicians, nurses, pharmacists, and social workers. Because Michigan Medicine is a teaching institution, notes are also created by hundreds of clinicians-in-training, including residents and fellows.

### Document index

As part of a larger Michigan Medicine-wide initiative to support improved access to the free text clinical notes for clinical care, operations, and research we developed a free text search engine, EMERSE [5], based on the open source Apache Lucene (https://lucene.apache.org) and Solr projects (http://lucene.apache.org/solr/). Solr creates an inverted index which makes it easy to identify all documents that contain specific words. Unlike some search engines, the index for EMERSE contains traditional stop words because many of these are also valid medical acronyms (e.g., IS: incentive spirometry; AND: axillary node dissection; OR: operating room). The standard Lucene tokenizer (StandardTokenizer) was used to tokenize the documents. As of December 2015 the index contained approximately 98.7 million documents and 12.7 billion words. In addition to the front-end user interface that EMERSE provides for standard users, the underlying Solr software includes a basic Query Screen interface that was used for the current analysis. This allowed us to search for single words and phrases, and quickly retrieve document counts without displaying any protected health information. Because no clinical notes were viewed by the team, this study was determined to be ‘not regulated’ by the University of Michigan Medical School Institutional Review Board.

### Search strategy

Using Solr, we obtained document counts for multiple variations in how numbers and other numerical concepts were expressed in the clinical notes, including the 12 types of lexical variants shown in Table 1. This included both Roman and Arabic numbers, as well as variations of numbers spelled out in words. Other numerical aspects that were explored included fractions, negative numbers, extremely large numbers, dimensions, dates, ages, tuples, and others. These lexical variants were not intended to be exhaustive of all possibilities, but were rather meant to represent common occurrences in the EHR based on clinical experience. We specifically included in our searches variations on commonly used numerical expressions and concepts that could be challenging to extract from the notes while preserving the meaning and context. All searches were case-insensitive and conducted using a lower-case index. Unless specified, the exact search strings used are those displayed in the tables in the Results section. Finally, to determine the potential impact of these numerical variations on tasks such as cohort identification, we used the EMERSE interface to obtain patient counts for 10 disorders and clinical findings that included either Roman or Arabic numerals. We compared the overlap between cohorts to determine how many patients would have been missed by searching for only one of the numeric variations but not the other (e.g., 3 vs III).

**Table 1:** Lexical Variants Included in this Paper

Lexical Variant Category	Examples
Positive integers	‘three’, ‘thirty-three’, ‘seventy-three’
Negative integers	‘minus three’, ‘minus 3’
Fractions	‘one third’, ‘one thirds’, ‘six eights’
Dimensions	‘one by three’, ‘two by four’
Ranges/odds	‘one to three’, ‘two to four’
Dates, including invalid	‘January 35’, ‘June 31’, ‘September 38’
Roman numerals	‘X’, ‘XV’, ‘XXIV’, ‘XXVIII’, ‘XXXV’
Medical classifications	‘1A’, ‘IID’, ‘type 2’, ‘type II’, ‘class III’
Ages, including implausible values	‘135 year old’ ‘septuagenarian’
Expressions of quantity	‘billions’, ‘octillion’, ‘gobs of’
Ordering/ranking	‘1st’, ‘1rd’, ‘firstly’, ‘1stly’, ‘primary’
Tuples	‘single’, ‘double’, ‘triple’, ‘quadruple’

## Results

The results from our number and numerical concept searches are presented in Tables 2, 3, 4, 5, 6, 7, 8, 9, 10, 11, 12, 13, 14, 15, 16, 17 and 18. All counts are presented as the number of distinct documents in which the terms appeared. Overall, we found substantial variation in how these numbers and concepts were expressed. Following is a brief overview of some notable findings from the tables. Table 2 demonstrates that negative numbers were represented in forms where the expression was completely spelled out (e.g., ‘minus five’) or with the spelled out ‘minus’ combined with Arabic numerals (e.g., ‘minus 5’). Fractions (e.g., ‘one-fifth’; Table 3), dimensions (e.g., ‘one by five’; Table 4), and ranges (e.g., ‘one to five’; Table 5) all appeared in spelled out forms.

**Table 2:** Negative Integers

minus one (821)	minus two (419)	minus three (218)	minus four (134)	minus five (129)	minus six (101)	minus seven (148)	minus eight (35)	minus nine (32)	minus ten (115)
minus 1 (2803)	minus 2 (2705)	minus 3 (1406)	minus 4 (631)	minus 5 (1643)	minus 6 (364)	minus 7 (948)	minus 8 (295)	minus 9 (202)	minus 10 (4453)
negative one (12,897)	negative two (3613)	negative three (1516)	negative four (980)	negative five (544)	negative six (622)	negative seven (329)	negative eight (263)	negative nine (203)	negative ten (5012)
negative 1 (97,662)	negative 2 (66,873)	negative 3 (54,088)	negative 4 (41,970)	negative 5 (40,719)	negative 6 (30,962)	negative 7 (26,100)	negative 8 (22,957)	negative 9 (20,923)	negative 10 (53,031)

**Table 3:** Fractions

	half(s)/halve(s)	third(s)	fourth(s)	fifth(s)	sixth(s)	seventh(s)	eighth(s)	ninth(s)	tenth(s)
one	287,671	57,040	4389	5454	177	48	1455	4	588
two	824	35,220	64	1112	6	21	9	1	182
three	2609	58	3347	286	6	19	287	0	91
four	1335	485	10	177	3	24	4	0	40
five	712	1	9	27	10	14	52	0	19
six	186	1	1	4	0	19	1	2	33
seven	89	0	0	7	0	0	33	0	19
eight	52	0	1	3	1	0	3	20	25
nine	36	0	0	0	0	0	1	0	48
ten	14	0	0	0	1	0	0	0	1

**Table 4:** Dimensions

	one	two	three	four	five	six	seven	eight	nine
one by	2332	12	7	1	1	2	0	1	0
two by	13	51	23	59	1	1	0	0	0
three by	1	8	20	8	5	0	0	0	0
four by	1	4	13	76	3	1	0	15	0
five by	0	3	2	5	5	1	1	1	1
six by	5	2	2	1	0	3	0	2	2
seven by	1	0	0	1	2	0	0	0	0
eight by	0	1	0	4	0	0	0	2	0
nine by	1	1	0	0	0	0	0	0	0
ten by	1	0	0	0	0	0	0	0	0

**Table 5:** Ranges or Odds

	one	two	three	four	five	six	seven	eight	nine
one to	24,976	599,217	25,720	5151	3848	3964	496	170	40
two to	493	2456	510,983	100,399	4602	3196	476	522	46
three to	91	206	651	363,750	41,499	25,572	1904	985	192
four to	55	63	90	176	125,943	2,284,611	1897	5972	99
five to	19	31	54	44	97	59,322	22,705	2157	353
six to	12	22	30	62	33	86	27,403	538,729	7200
seven to	3	6	10	16	13	25	65	15,433	1650
eight to	12	5	9	15	20	28	12	41	8379
nine to	8	3	5	3	17	15	5	2	27
ten to	18	17	13	14	20	10	17	9	9

**Table 6:** Invalid Dates^a^

	31	32	33	34	35	36	37	38	39
January	55,596^b^	7	11	3	11	6	3	5	8
February	30	5	6	2	4	1	5	0	3
March	56,701^b^	23	7	12	113	1	12	9	5
April	285	6	8	4	4	0	4	2	8
May	50,884^b^	19	9	18	4	4	16	8	11
June	31	273	10	5	6	5	3	5	15
July	59,207^b^	9	7	11	7	8	4	1	3
August	57,896^b^	5	10	6	8	8	5	5	7
September	257	6	0	5	6	4	1	4	5
October	59,150^b^	13	10	4	2	3	5	5	3
November	234	6	2	3	10	7	1	5	3
December	25,840^b^	7	10	6	2	3	2	4	3

^a^The cell in the upper right corner would be ‘January 39’. Not included in this table is ‘February 30’ which appeared in 117 documents. Total number of invalid date instances in this table: 1917

^b^ The 31st day for January, March, May, July, August, October, and December are, of course, valid

**Table 7:** Roman Numerals

	I (34,856,243)	II (4,814,592)	III (3,467,400)	IIII (487)	IIIII (62)	IIIIII (5)	IIIIIII (3)	IIIIIIII (2)	IIIIIIIII (1)
				IV (9,375,039)	V (4,420,994)	VI (577,732)	VII (171,958)	VIII (85,330)	IX (47,108)
X (15,589,182)	XI (27,201)	XII (1,105,852)	XIII (2449)	XIV (511)	XV (2577)	XVI (22)	XVII (28)	XVIII (19)	XIX (19)
XX (104,180)	XXI (244)	XXII (154)	XXIII (2)	XXIV (4)	XXV (2)	XXVI (3)	XXVII (1)	XXVIII (0)	XXIX (0)
XXX (8856)	XXXI (1)	XXXII (0)	XXXIII (0)	XXXIV (0)	XXXV (0)	XXXVI (0)	XXXVII (0)	XXXVIII (0)	XXXIX (2)

**Table 8:** Medical Categorizations^a^

	A	B	C	D	E	F	G	H	I	J
1	298,397	162,822	92,512	64,856	49,791	40,990	223,638	173,504	17,135	15,441
2	143,858	70,087	29,521	335,947	15,212	18,362	219,114	156,211	3232	2898
3	66,477	27,332	24,692	314,058	14,396	14,528	55,856	147,656	1874	1714
4	171,463	159,144	138,104	33,191	12,352	19,792	58,001	217,040	1146	1081
5	194,432	93,058	151,822	101,684	14,428	34,077	130,574	149,902	673	946
I	93,721	75,347	159,150	13,964,384	497,302	27,699,212	39,540	45,987	4,814,592	434,416
II	56,631	43,207	4846	274	372	2500	53	2158	3,467,400	2
III	65,347	45,687	33,381	60	97	9	5	21	487	2
IV	41,830	15,552	509,947	2695	40,328	90,9986	576	62,302	533	108
V	295,868	54,862	103,848	9929	158,751	106,698	9271	595,776	577,732	328

^a^The term in the upper left would be ‘1A’. These are often used in classifying disorders such as Hyperlipoproteinemia Type IIA or Stage 3B Lung Cancer. Note that some of the terms with Roman numerals could be confused with other medical abbreviations (e.g., *VA* Veterans Affairs, *1G* 1 g, *3D* Three-dimensional, *IC* Intracardiac, *ID* Infectious diseases). IF is a common English word (case sensitive searches were not conducted for this analysis)

**Table 9:** Additional Categorization Variations^a^

	1	I	2	II	3	III	4	IV	IIII	5	V
type	674,898	231,183	1,588,852	421,332	196,961	47,794	167,557	15,068	5	161,395	1673
phase	88,407	39,641	125,204	53,863	36,978	8975	1750	431	1	28,526	61
grade	639,287	184,486	426,407	155,115	221,568	94,407	55,841	30,020	23	20,740	5251
stage	149,938	357,732	169,038	273,244	332,2767	274,993	90,336	285,535	31	36,419	55,780
class	72,731	298,391	94,568	173,749	112,243	128,196	27,082	36,450	26	36,759	5707
score	171,243	15,607	107,100	266	121,064	246	100,209	133	0	112,719	100

^a^Additional variations in how some categorizations in medicine are represented with either Arabic or Roman numerals. The cell in the upper right hand corner represents ‘type V’ whereas the lower left is ‘score 1’

**Table 10:** Diabetes Terminology Variations

Phrase	n
Type I diabetes	41,007
Type II diabetes	109,739
Type III diabetes	6
Type IV diabetes	8
TIDM	607
TIIDM	992
Type III DM	2
Type IV DM	1
T1DM	12,725
T2DM	70,314
T21DM	5
T12DM	2
Type 1 diabetes	271,541
Type 2 diabetes	871,228
Type 21 diabetes	4
Type 12 diabetes	2
DM1	17,166
DM 1	7238
DM2	167,534
DM 2	25,407
DMI	79,253
DM I	8317
DMII	56,942
DM II	44,983

**Table 11:** Biologically Implausible Ages

Phrase	n
123 year old	3
124 year old	1
125 year old	22
126 year old	2
127 year old	4
128 year old	2
129 year old	2
130 year old	55
131 year old	1
132 year old	2
133 year old	2
134 year old	3
135 year old	4
136 year old	2
137 year old	29
138 year old	4
139 year old	1
140 year old	29
150 year old	128
160 year old	13
170 year old	3
180 year old	5
190 year old	3
200 year old	23

**Table 12:** Age Groups by Decade

Phrase	n
quinquagenarian	0
sexagenarian	1
septuagenarian	112
octogenarian	239
nonagenarian	45
centenarian	16
supercentenarian	0

**Table 13:** Ordering and Ranking^a^

	st	nd	rd	th
1	862,447^b^	79	7	299
2	282	801,375^b^	360	270
3	27	617	626,822^b^	694
4	17	46	432	442,238^b^
5	16	16	54	481,412^b^

^a^Ways in which ordering and ranking is described. As an example, the cell in the upper right corner is the term ‘1th’

^b^ Cells containing valid expressions

**Table 14:** Very Large and Small Quantities

Phrase	n
minus infinity	0
negative infinity	2
hundred	17,760
hundreds	9215
thousand	14,917
thousands	6401
hundred thousand	146
million	75,013
millions	1179
billion	46,081
billions	381
trillion	51
trillions	27
quadrillion	2
quadrillions	1
octillion	3
nonillion	2
undecillion	1
googolplex	0
googol	0
infinity	6325

**Table 15:** Imprecise and Informal Expressions of Quantity

Phrase	n
couple of	1673,735
lots of	328,506
not much	113,336
few of	35,803
small number of	12,358
hundreds of	7371
all kinds of	6940
thousands of	4611
tons of	3018
too many to count	1346
massive amounts of	1187
very small number of	1104
far more than	971
way more than	820
very large number of	623
millions of	561
way too many	364
huge number of	260
gobs of	199
vanishingly small	179
uncountable	133
hell of a lot	69
lion’s share of	67
vast quantities of	48
waist deep in	24
infinitesimally small	23
tiny number of	19
infinitely more	17
miniscule amounts of	14
gazillion	12
crap load of	8
shit load	7
up the wazoo	6
infinitely small	6
bazillion	5
infinitely less	3
infinitely large	3
butt load	3
boat loads of	3
buttload	1

**Table 16:** Additional Ways in Which Ordering and Ranking are Described

first (7,172,197)	firstly (5690)	1stly (0)	primary (10,994,471)	1ary (26)
second (3,576,368)	secondly (33,662)	2ndly (26)	secondary (5,630,281)	2ndary (3249)
third (1,317,624)	thirdly (5716)	3rdly (2)	tertiary (35,083)	3rdary (0)
fourth (538,499)	fourthly (301)	4thly (0)	quaternary (377)	
fifth (473,144)	fifthly (40)	5thly (0)	quinary (4)	
sixth (124,807)	sixthly (6)	6thly (0)	senary (2)	
seventh (77,463)	seventhly (0)	7thly (0)	septenary (0)	
hundredth (40)				
thousandth (168)			unary (10)	2ary (315)
millionth (12)			binary (1367)	3ary (2)
billionth (3)			ternary (6)	4ary (0)

**Table 17:** Tuples

singling (242)	singled (1362)	singles (6621)	single (4,429,544)	singleton (58,421)
doubling (24,555)	doubled (49735)	doubles (5467)	double (1179,932)	twins (90,512)
tripling (819)	tripled (2806)	triples (533)	triple (338,340)	triplets (46,831)
quadrupling (85)	quadrupled (445)	quadruples (11)	quadruple (14,966)	quadruplets (828)
quintupling (1)	quintupled (4)	quintuples (1)	quintuple (996)	quintuplets(122)
sextupling (0)	sextupled (1)	sextuples (0)	sextuple (9)	sextuplets (13)
septupling (0)	septupled (0)	septuples (0)	septuple (2)	septuplets (5)
octupling (0)	octupled (0)	octuples (0)	octuple (1)	octuplets (0)

**Table 18:** Results from a Cohort Identification Experiment^a^

(a)	(b)	(c)	(d)	(e)	(f)	(g)
Phrase 1 (containing the Arabic numerical variant)	Number of patients with Phrase 1 only	% of patients *missed* if searching only for Phrase 1	Number of patients with both Phrase 1 and Phrase 2	Number of patients with Phrase 2 only	% of patients *missed* if searching only for Phrase 2	Phrase 2 (containing the Roman numerical variant)
citrullinemia type 1	2	25.0	1	1	50.0	citrullinemia type I
type 2 diabetes mellitus	43,777	10.5	7919	6053	75.8^b^	type II diabetes mellitus
type 1 neurofibromatosis	181	24.5	56	77	57.6^b^	type I neurofibromatosis
Tanner Stage 3	7639	57.8^b^	1373	12,367	35.7	Tanner Stage III
grade 3 anaplastic astrocytoma	42	36.7	27	40	38.5	grade III anaplastic astrocytoma
stage 3 chronic kidney disease	615	67.4^b^	446	2190	18.9	stage III chronic kidney disease
factor 9 deficiency	14	68.1^b^	51	139	6.9	factor IX deficiency
class 3 malocclusion	135	81.2^b^	115	1079	10.2	class III malocclusion
phase 1 clinical trial	320	66.5^b^	263	1158	18.4	phase I clinical trial
Mallampati score: 4	121	27.8	1	47	71.6^b^	Mallampati score: IV

^a^Reesults from a cohort identification exercise for 10 diagnoses and clinical findings in the clinical notes, including counts of the number of patients identified by searching for phrases containing either the Arabic or Roman numeral variants, or both. The percentage of patients potentially missed by searching for only one of the variants is displayed

^b^ Cells with percentages > 50%

Invalid dates such as ‘January 39’ (Table 6) appeared with low frequency, but were still present for nearly all of the combinations for which we searched. Roman numerals (Table 7) were also present in the documents, although the frequency trailed off substantially beyond 30 (‘XXX’). There were a small number of documents that also contained incorrectly formed Roman numerals such as ‘IIII’ rather than ‘IV’. Tables 8 and 9 show variations in how some concepts related to medical scoring, staging, grading, and other clinical classifications were recorded, including variations using both Roman and Arabic numbers. Differences were noted in the frequency in how these numbers were used. For example, with ‘type’ (e.g., ‘type 2’ vs. ‘type II’) use of the Arabic numeral was more frequent than use of the Roman numerals. By contrast, with ‘class’ (e.g., ‘class 2’ vs. ‘class II’) the Roman numerals were more common than the Arabic numerals except for ‘Class 5’. Table 10 displays similar examples of variations for diabetes. Table 10 also illustrates some of the typographic errors that exist in the notes (e.g., ‘type 21 diabetes’), albeit at low frequencies.

Table 11 shows biologically implausible ages, starting at ‘123 year old’. Note that the oldest living person in recorded history lived to 122 years [22]. Table 12 reports on ages described by decades. The most commonly used term was ‘octogenarian’, followed by ‘septuagenarian’. Table 13 shows how ranking is sometimes represented, including variations that were both correct (e.g., ‘1st’ and ‘3rd’) and incorrect (e.g., ‘1rd’ and ‘3st’). These suffixes also existed with dates, including ‘June 31st’ which appeared 29 times and ‘November 31st’ which appeared 11 times, neither of which are valid dates. Table 14 displays very large and very small quantities, expressed as spelled out words. While no document included ‘googolplex’, a finite number of documents (*n* = 6325) used ‘infinity’, and a very small number (*n* = 2) included the very small number ‘negative infinity’. Imprecise and informal expressions of quantity are reported in Table 15. Terms and phrases that appeared in a small subset of documents included ‘gobs of’, ‘gazillion’, and ‘bazillion’. Other ordering and ranking variations are listed in Table 16, and tuples such as ‘doubled’ and ‘quadruplets’ are reported in Table 17.

Table 18 displays examples showing the real-world implications of not considering the numeric variations in the clinical notes. This table reports on the number of patients having phrases in their notes representing diagnoses and clinical findings that could be used for cohort identification. These phrases contain either an Arabic numeral (column a) or a Roman numeral (column g). Column (b) displays the number of patients who had only the phrase with the Arabic numeral variant among all of their notes, whereas column (f) displays the number of patients who had only the phrase with the Roman numeral variant in their notes. Column (d) shows the number of patients that had both variants in their notes. For patients in column (d), searching for either variant (containing Arabic or Roman numbers) would be sufficient to identify the patient. Column (c) reports on the percentage of patients that would have been missed had only the Arabic numeral variant been used in the search, whereas column (e) represents the percentage that would have been missed if only the Roman numeral variant had been used in the search.

## Discussion

This work demonstrates the substantial variability in how numbers and other numerical concepts are represented in clinical notes derived from both a home-grown and a vendor EHR system. This variability was not only a result of normal English language variations, but of typographic errors [23] as well as incorrect usage errors. Our findings highlight data quality issues that could impact the performance of information retrieval and extraction systems, and demonstrates the complexity of medical information containing numbers and numerical concepts.

Importantly, this study also shows how much these variations could impact research endeavors such as cohort identification. Among the 10 examples shown in Table 18, eight of them resulted in more than 50% of the patients being missed under the scenario of searching for a phrase with only the Arabic or Roman numerals but not both variations. For the case of ‘class 3 malocclusion’ more than 80% of cases would have been missed if ‘class III malocclusion’ was excluded from the search. Interestingly, a search for ‘grade 3 anaplastic astrocytoma’ revealed a patient count of 69 whereas a similar search for ‘grade III anaplastic astrocytoma’ revealed a count of 67. This might lead one to conclude that approximately 68 such patients existed in the data set. However, our analysis revealed little overlap (*n* = 27) between these two sets, with 109 total patients identified when both variations were included. In many real-life cohort identification tasks, structured data such as International Classification of Disease, version 10 (ICD-10) codes may also used in addition to, or even instead of the free text, but such codes are known to be unreliable in certain contexts [24].

The frequencies reported in this paper were not meant to provide insights about whether they were the ‘expected’ number of instances but rather to show how many of these exist in the clinical notes. Any count above zero means that an information extraction process would have to consider that variation or it could be missed. However, one insight that can be drawn from the frequencies includes cases in which some counts appear higher than their neighbors. This could imply a dual use of the concept in which case disambiguation would be needed. For example, the number of instances of the Roman numeral ‘IV’ was nearly three times the frequency of ‘III’ and two times the frequency of ‘V’. Since ‘IV’ is a commonly used abbreviation for ‘intravenous’, this is a likely explanation for that observation. Many of the abnormal and unusual representations were rare considering how many documents were included in the full dataset. While this is reassuring for those conducting research or surveillance at a population level, the invalid or inappropriate use of numbering could have a more meaningful impact at an individual patient level, where a mistakenly interpreted or overlooked numerical concept could result in improper treatment decisions.

These findings also highlight the importance of taking into account the potential for both predictable and non-standard variations with tasks such as natural language processing, information extraction, or query expansion in information retrieval systems. It is also worth noting that the low frequency of some findings may mean that comparable examples do not exist in the document corpora used for NLP training tasks such as those used for the i2b2 challenge competitions [25]. This work could also inform ways in which data entry systems could be designed to identify these errors or variants to encourage users to enter more appropriate or standard terms.

It is possible that some of these complexities could be resolved by ‘normalizing’ the variations to a common form in a pre-processing step (e.g., converting ‘VI’ to 6). Indeed, some tools such as cTAKES [26] already does some of this work. Yet disambiguation may also be necessary since many of the concepts can appear in contexts beyond standard numbers. For example, ‘I’ could be the Roman numeral 1, or the common pronoun. The phrase ‘2/2’ could be ‘2 out of 2’, ‘secondary to’, or even ‘February 2’. Word sense disambiguation continues to be an active area of NLP research [10, 27, 28]. Information extraction system designers must also consider how to handle values that are invalid such as out-of-range ages (e.g., ‘135 year old’) rather than simply ignoring them. Terms like ‘octogenarian’, and especially ‘nonagenarian’ can reveal a patients approximate age and thus should be taken into consideration when building or customizing de-identification systems.

Invalid dates (e.g., ‘March 35’) also represent a challenge. Many programming languages (e.g., Java) by default handle invalid dates in a lenient manner, meaning that a date such as ‘March 35’ would be converted to April 4. Care must also be taken when considering the interpretation of negative numbers. Depending on tokenization, a system might identify a number ‘1’ or ‘one’ but miss the ‘negative’ qualifier in front of it if it is written as ‘negative 1’ or ‘minus one’ as opposed to ‘-1’. Tools do exist to help with number normalization, [29, 30] and these should be considered when processing clinical text. Other tools have been developed to identify various concepts related to numbering including for Time (MedTime) [31] as well as cancer staging (e.g., ‘Stage III lung cancer’) and dimensions (MedKATp) [32]. Tokenization may also be important. A technical report about tokenization of MEDLINE abstracts briefly discusses how various tokenizers handle text including fractions [33]. A more recent paper noted the lack of focus on biomedical tokenization [34].

The issues described here are related to both semantic and syntactic heterogeneity, and are contributing factors limiting the widespread semantic interoperability of EHR data [35–37]. In some cases simple normalization to a canonical form should be easily achievable. In other cases, however, the complexities of natural language introduce challenges that will require additional work including disambiguation, intelligent tokenization, and sophisticated processing (e.g., machine learning). It will be important for those working with the free text data to understand the text being analyzed and have plans for how outlier situations (e.g., invalid dates) will be handled. It will also be important to utilize vocabularies or ontologies with broad coverage of synonyms, near synonyms, and lexical variants. For example, ‘TIIDM’ appeared in nearly 1000 notes in our dataset but that term variant for ‘type 2 diabetes mellitus’ is not present in the Unified Medical Language System (UMLS), whereas ‘T2DM’ is in UMLS.

Additional complexities not analyzed in the current work included variations in units, which can further complicate information extraction. For example, weights can be written as “pounds”, “lbs”, “lb”, “#”, and sometimes no unit might be provided, meaning that additional work would be needed to determine if English (pounds) or metric (kg) weights were being described.

It is also worth noting that these data quality and normalization issues are not unique to clinical notes derived from EHRs. For example, the incorrect ‘3nd’ (as opposed to the correct ‘3rd’) appears in PubMed abstracts [38, 39] as well as in clinical trial descriptions listed on ClinicalTrials.gov [40, 41]. Even terms such as ‘octogenarian’ [42] and ‘nonagenarian’ [43] appear on ClinicalTrials.gov. Indeed, recent work has suggested formal representations for numeric data in clinical trial reports to aid in interpretation of the results [44]. Variability can also be found when identifying concepts within the UMLS Terminology Services Metathesaurus Browser (https://uts.nlm.nih.gov/metathesaurus.html). For example, as of July 2018, searching for the term ‘stage 3’ yields 233 results whereas searching for ‘stage III’ yields 803 results. Even ‘type IIII’ (an invalid form of the Roman numeral ‘IV’) appears in a UMLS entry (CUI C2612864), which is likely a typographic error.

Our work has several limitations. First, this study was conducted at a single site, and other medical centers or EHRs may contain different types or frequencies of variations that we did not detect. Second, we quantified only a subset of possible variations. For example, we did not explore the frequency of spelling errors such as ‘sevin’, and there are other types of variations which were not included due to space limitations. Third, the frequency of some of the term variants we identified could be falsely elevated due to copy-pasting of text between notes. Nevertheless the tables we present in this work show a wide variety of possible ways in which numbers and numerical concepts are actually represented in the clinical EHR notes. Fourth, it may be the case that many of these variations would have no clinical significance with information extraction tasks. We believe, however, that it is difficult to generalize about what types of information are clinically significant versus insignificant as this may depend heavily on the specific information needs of users.

## Conclusions

As precision medicine and personalized healthcare become more prevalent, computers might be tasked with making automatic decisions or recommendations on an individual patient basis using the information found within EHR notes. Thus, there could be a direct effect on patient outcomes if information is interpreted incorrectly or overlooked. Further, the present study shows that these variations could have direct impact on cohort identification tasks unless care is taken to ensure search strings inclusive of the existing variations. Until then, clinicians and informaticians seeking to use these data should consider the variations described in this paper when designing strategies to ensure that information extraction tasks and systems are as accurate as possible.

## Abbreviations

EHR: Electronic health record
NLP: Natural language processing
UMLS: Unified Medical Language System

## References

[1] Adler-Milstein J, DesRoches CM, Kralovec P, Foster G, Worzala C, Charles D, Searcy T, Jha AK (2015). Electronic health record adoption in US hospitals: Progress continues, but challenges persist. Health Aff (Millwood).

[2] Kahn MG, Weng C (2012). Clinical research informatics: a conceptual perspective. J Am Med Inform Assoc.

[3] Friedman C, Rindflesch TC, Corn M (2013). Natural language processing: state of the art and prospects for significant progress, a workshop sponsored by the National Library of medicine. J Biomed Inform.

[4] Biron P, Metzger MH, Pezet C, Sebban C, Barthuet E, Durand T (2014). An information retrieval system for computerized patient records in the context of a daily hospital practice: the example of the Leon Berard Cancer center (France). Appl Clin Inform.

[5] Hanauer DA, Mei Q, Law J, Khanna R, Zheng K (2015). Supporting information retrieval from electronic health records: a report of University of Michigan's nine-year experience in developing and using the electronic medical record search engine (EMERSE). J Biomed Inform.

[6] Koopman B, Bruza P, Sitbon L, Lawley M (2012). Towards semantic search and inference in electronic medical records: an approach using concept--based information retrieval. Australas Med J.

[7] Edinger T, Cohen AM, Bedrick S, Ambert K, Hersh W (2012). Barriers to retrieving patient information from electronic health record data: failure analysis from the TREC medical records track. AMIA Annu Symp Proc.

[8] Nadkarni PM, Ohno-Machado L, Chapman WW (2011). Natural language processing: an introduction. J Am Med Inform Assoc.

[9] Ruch P, Baud R, Geissbuhler A (2003). Using lexical disambiguation and named-entity recognition to improve spelling correction in the electronic patient record. Artif Intell Med.

[10] McInnes BT, Stevenson M (2014). Determining the difficulty of word sense disambiguation. J Biomed Inform.

[11] Moon S, Pakhomov S, Melton GB (2012). Automated disambiguation of acronyms and abbreviations in clinical texts: window and training size considerations. AMIA Annu Symp Proc.

[12] Wu Y, Denny JC, Rosenbloom ST, Miller RA, Giuse DA, Song M, Xu H (2015). A preliminary study of clinical abbreviation disambiguation in real time. Appl Clin Inform.

[13] Grouin C, Grabar N, Hamon T, Rosset S, Tannier X, Zweigenbaum P (2013). Eventual situations for timeline extraction from clinical reports. J Am Med Inform Assoc.

[14] Kovacevic A, Dehghan A, Filannino M, Keane JA, Nenadic G (2013). Combining rules and machine learning for extraction of temporal expressions and events from clinical narratives. J Am Med Inform Assoc.

[15] Roberts K, Rink B, Harabagiu SM (2013). A flexible framework for recognizing events, temporal expressions, and temporal relations in clinical text. J Am Med Inform Assoc.

[16] Hanauer DA, Liu Y, Mei Q, Manion FJ, Balis UJ, Zheng K (2012). Hedging their mets: the use of uncertainty terms in clinical documents and its potential implications when sharing the documents with patients. AMIA Annu Symp Proc.

[17] Hripcsak G, Albers DJ (2013). Next-generation phenotyping of electronic health records. J Am Med Inform Assoc.

[18] Overby CL, Pathak J, Gottesman O, Haerian K, Perotte A, Murphy S, Bruce K, Johnson S, Talwalkar J, Shen Y (2013). A collaborative approach to developing an electronic health record phenotyping algorithm for drug-induced liver injury. J Am Med Inform Assoc.

[19] Pathak J, Kho AN, Denny JC (2013). Electronic health records-driven phenotyping: challenges, recent advances, and perspectives. J Am Med Inform Assoc.

[20] Shivade C, Raghavan P, Fosler-Lussier E, Embi PJ, Elhadad N, Johnson SB, Lai AM (2014). A review of approaches to identifying patient phenotype cohorts using electronic health records. J Am Med Inform Assoc.

[21] Weiskopf NG, Hripcsak G, Swaminathan S, Weng C (2013). Defining and measuring completeness of electronic health records for secondary use. J Biomed Inform.

[22] Whitney CR. Jeanne Calment, World’s elder, dies at 122. New York Times; 1997. Found at: https://www.nytimes.com/1997/08/05/world/jeanne-calment-world-s-elder-dies-at-122.html. Accessed 4 Mar 2019.

[23] Wu X, Wu C, Zhang K, Wei D (2016). Residents’ numeric inputting error in computerized physician order entry prescription. Int J Med Inform.

[24] Rhodes ET, Laffel LM, Gonzalez TV, Ludwig DS (2007). Accuracy of administrative coding for type 2 diabetes in children, adolescents, and young adults. Diabetes Care.

[25] Uzuner O, Bodnari A, Shen S, Forbush T, Pestian J, South BR (2012). Evaluating the state of the art in coreference resolution for electronic medical records. J Am Med Inform Assoc.

[26] Savova GK, Masanz JJ, Ogren PV, Zheng J, Sohn S, Kipper-Schuler KC, Chute CG (2010). Mayo clinical text analysis and knowledge extraction system (cTAKES): architecture, component evaluation and applications. J Am Med Inform Assoc.

[27] Chasin R, Rumshisky A, Uzuner O, Szolovits P (2014). Word sense disambiguation in the clinical domain: a comparison of knowledge-rich and knowledge-poor unsupervised methods. J Am Med Inform Assoc.

[28] Garla VN, Brandt C (2013). Knowledge-based biomedical word sense disambiguation: an evaluation and application to clinical document classification. J Am Med Inform Assoc.

[29] Java Number Normalizer (Beta), Natural language processing lab, bar-Ilan University. http://u.cs.biu.ac.il/~nlp/resources/downloads/java-number-normalizer-beta/. Accessed 2 Feb 2019.

[30] Class NumberNormalizer, Stanford University. http://nlp.stanford.edu/nlp/javadoc/javanlp/edu/stanford/nlp/ie/NumberNormalizer.html. Accessed 2 Feb 2019.

[31] MedTime Project Page. http://ohnlp.org/index.php/MedTime_Project_Page. Accessed 2 Feb 2019.

[32] MedKATp. http://ohnlp.org/index.php/MedKATp. Accessed 2 Feb 2019.

[33] A Comparison of 13 Tokenizers on MEDLINE. https://lhncbc.nlm.nih.gov/publication/lhncbc-tr-2006-003. Accessed 2 Feb 2019.

[34] Cruz Díaz NP, Maña López MM. An analysis of biomedical tokenization: problems and strategies. In: Proceedings of the Sixth International Workshop on Health Text Mining and Information Analysis (Louhi). Lisbon; 2015. p. 40–9. Found at: https://aclweb.org/anthology/W/W15/W15-2605.pdf. Accessed 4 Mar 2019.

[35] Berges I, Bermudez J, Illarramendi A (2012). Toward semantic interoperability of electronic health records. IEEE Trans Inf Technol Biomed.

[36] Martinez-Costa C, Cornet R, Karlsson D, Schulz S, Kalra D (2015). Semantic enrichment of clinical models towards semantic interoperability. The heart failure summary use case. J Am Med Inform Assoc.

[37] Tapuria A, Kalra D, Kobayashi S (2013). Contribution of clinical archetypes, and the challenges, towards achieving semantic interoperability for EHRs. Healthc Inform Res.

[38] Kokcu A, Tosun M, Alper T, Sakinci M (2011). Primary carcinoma of the neovagina: a case report. Eur J Gynaecol Oncol.

[39] Sayyah M, Boostani H, Ashrafpoori M, Pakseresht S (2015). Effects of atorvastatin on negative sign in chronic schizophrenia: a double blind clinical trial. Iran J Pharm Res.

[40] Stimulated Intrauterine Insemination Cycles and Unstimulated Intrauterine Insemination Cycles in Couples With Unexplained Infertility. https://clinicaltrials.gov/ct2/show/NCT02461173. Accessed 2 Feb 2019.

[41] East-West Collaboration Treatment Using Bee Venom Acupuncture and NSAIDs for Chronic Cervicalgia. https://clinicaltrials.gov/ct2/show/NCT01922466. Accessed 2 Feb 2019.

[42] Direct Oral Anticoagulants Pharmacodynamics in Octogenarian Patients With Atrial Fibrillation. https://clinicaltrials.gov/ct2/show/NCT02623049. Accessed 2 Feb 2019.

[43] Revascularization in Nonagenarian Patients With Critical Lower Limb Ischaemia (NONA-CLI). https://clinicaltrials.gov/ct2/show/NCT02517840. Accessed 2 Feb 2019.

[44] Tong M, Hsu W, Taira RK (2013). A formal representation for numerical data presented in published clinical trial reports. Stud Health Technol Inform.

